# Prognostic Role of Soluble Programmed Death Ligand 1 in Non-Small Cell Lung Cancer: A Systematic Review and Meta-Analysis

**DOI:** 10.3389/fonc.2021.774131

**Published:** 2021-12-23

**Authors:** Guixiang Liao, Zhihong Zhao, Yuting Qian, Xiean Ling, Shanyi Chen, Xianming Li, Feng-Ming (Spring) Kong

**Affiliations:** ^1^ Department of Radiation Oncology, Shenzhen People’s Hospital, The Second Clinical Medical College, Jinan University, Shenzhen, China; ^2^ Department of Nephrology, Shenzhen People’s Hospital, The Second Clinical Medical College, Jinan University, Shenzhen, China; ^3^ Department of Thoracic Surgery, Shenzhen People’s Hospital, The Second Clinical Medical College, Jinan University, Shenzhen, China; ^4^ Department of Clinical Oncology, The University of Hong Kong–Shenzhen Hospital, Shenzhen, China; ^5^ Department of Clinical Oncology, Li Ka Shing Faculty of Medicine, The University of Hong Kong, Hong Kong, China

**Keywords:** overall survival, prognosis, soluble programmed death ligand 1, immunotherapy, non-small cell lung cancer, immune checkpoint inhibitors

## Abstract

**Objective:**

The objective of this study was to explore whether soluble programmed death ligand 1 (sPD-L1) is a potential prognostic biomarker in patients with non-small cell lung cancer (NSCLC).

**Methods:**

A comprehensive search of electronic databases was carried out. Original studies with inclusion of sPD-L1, progression-free survival, and overall survival in NSCLC were eligible. The primary endpoints were overall survival and progression-free survival. Hazard ratios (HRs) and 95% confidence intervals (CIs) were applied for data analysis.

**Results:**

Eight studies involving 710 patients with NSCLC were included in the analysis. A pooled data analysis revealed that high levels of sPD-L1 were correlated with poorer overall survival (HR = 2.34; 95% CI = 1.82–3.00; *P* < 0.001) and progression-free survival (HR = 2.35; 95% CI = 1.62–3.40, *P* < 0.001). A subgroup analysis revealed that high levels of sPD-L1 were correlated with poor overall survival in patients treated with immunotherapy (HR = 2.40; 95% CI = 1.79–3.22; *P* < 0.001).

**Conclusion:**

This pooled analysis of published data suggests that sPD-L1 may serve as a readily available biomarker for survival in NSCLC patients treated with ICI based treatment. Prospective studies with well-designed standard assessment methods should be conducted to validate the prognostic role of sPD-L1 in NSCLC.

**Systematic Review Registration:**

https://www.crd.york.ac.uk/prospero/display_record.php?ID=CRD42021283177.

## Introduction

Lung carcinoma is the most aggressive cancer worldwide (1). Approximately 85% of lung carcinomas are non-small cell lung cancers (NSCLC) (2). Accumulating evidence suggests that programmed death 1 receptor (PD-1) and its ligand, programmed death ligand 1 (PD-L1), are upregulated in lung cancers (3). Inhibition of the PD-1 and PD-L1 pathways are novel targets for immunotherapy, which has improved the outcomes of lung cancer (4).

PD-L1 is expressed in different types of cancer (5); membrane-bound PD-L1 is regarded as a prognostic factor in lung cancer (6, 7). However, apart from tumor tissue biomarkers, some blood-based biomarkers have been reported as valuable biomarkers (8, 9). In fact, blood tests have the benefits of being minimally invasive and allow monitoring of the ongoing treatment (10). Notably, both PD-1 and PD-L1 can exist either as membrane-bound or soluble form (11, 12). Some studies have reported that soluble PD-L1 (sPD-L1) can be detected in the blood of patients with cancer and is regarded as a prognostic marker (13–15). Although the source of sPD-L1 remains elusive, data from NSCLC favors the point that the proteins are derived from cancer cells (16). While the functions of sPD-L1 remain unclear, several biological effects have been proposed (17). Tumor cell-derived sPD-L1 has been suggested to induce apoptosis in T cells in patients with advanced renal carcinoma (18). SPD-L1 has also been hypothesized to inactivate the circulating tumoricidal T cells, thereby reducing antitumor immune activity. Furthermore, sPD-L1 can compete and saturate PD-1 binding sites, thereby eluding the activity of anti-PD-1 agents (16). Other study has indicated that sPD-L1 can promote Th1/Th17 cell proliferation (19).

PD-L1 can be divided into membrane-bound PD-L1 and sPD-L1 (17), and the detection of sPD-L1 in the plasma of cancer patients has gained great interest among researchers. Interestingly, recent studies have indicated that sPD-L1 may be a prognostic factor in multiple types of cancers (20–24). Previous meta-analyses have suggested that sPD-L1 can predict OS by combining data from various types of cancers (25–27). However, the prognostic value of sPD-L1 in several types of cancers has conflicting results (17, 28, 29). Zheng et al. (28) reported that patients with gastric cancer and with a higher sPD-L1 level had better overall survival (OS) than those patients with a low sPD-L1 level. Zhang et al. (29) indicated that a higher sPD-L1 level had a poor prognosis in patients with lung cancer. However, the predictive role of sPDL1 in NSCLC remains unknown. Moreover, whether sPD-L1 could be a prognostic factor in patients with NSCLC receiving immune checkpoint inhibitors (ICIs) is not clear. This study aimed to conduct a systematic review and meta-analysis to study these questions.

## Methods

### Literature Search

This systematic review was conducted according to the Preferred Reporting Items for Systematic Reviews and Meta-analyses (PRISMA) (30). The protocol was registered on PROSPERO: CRD4202128377. Electronic databases from PubMed, Web of Science, EMBASE, and Cochrane library were searched to identify studies that evaluated the prognostic role of sPD-L1 in NSCLC. The following keywords were applied: cancer, carcinoma, tumor, or neoplasm; serum, plasma, blood serum, blood, circulating, or soluble; sPD-L1 or B7-H1 or PD-L1; survival or predictive or prognosis or prognostic, and non-small cell lung cancer. The latest search was conducted on October 1, 2021. Furthermore, the references of the included studies were screened for missing studies that potentially met the inclusion criteria. Two independent reviewers (GL and ZZ) performed the study selection.

### Inclusion and Exclusion Criteria for Meta-Analysis

The inclusion criteria were as follows: (a) patients were with NSCLC, (b) the sPD-L1 levels were analyzed in serum or plasma, (c) the study was reported in a full research publication in English, (d) the relationship with human survival outcomes (overall survival, OS, or progression-free survival, PFS) was determined, and (e) the number of included cases was not less than 20. The exclusion criteria were as follows (27): (a) comments, systematic reviews, case reports, animal studies, and studies without sufficient data for meta-analysis were excluded and (b) studies with survival outcomes provided with survival cure and with the precision HR cannot be calculated were excluded.

### Data Extraction

Two independent reviewers (GL and ZZ) extracted information by reviewing the eligible studies. The extracted data were as follows: the first author, the year of publication, the country where the study originated from, cancer type, sample size, age, study design, cutoff value of sPD-L1, Eastern Cooperative Oncology Group performance status, smoking status, lines of ICI treatment, tissue PD-L1 tumor proportion score, follow-up time, survival outcomes with regard to high/low sPD-L1 levels, and the relationship between clinicopathologic features and sPD-L1 concentrations.

### Quality Assessment

The quality of the included studies was evaluated according to the Newcastle–Ottawa Quality Assessment Scale (NOS) (31). The scores were given from 0 to 9, according to the quality of the studies. A score equal to or higher than seven was regarded as high quality. Quality assessment was performed GL and ZZ. Any disagreements were resolved by a discussion with the author group.

### Statistical Analysis

The association between sPD-L1 and survival outcomes was measured with hazard ratios (HRs) and 95% confidence interval (CI). Review Manage (5.4 version) (Cochrane Centre) was used. Furthermore, random-effect model was applied. Heterogeneity was evaluated using *I* ^2^ (32, 33). Subgroup analyses were conducted based on Asian and non-Asian populations, the year of publication, sample size, cutoff values, study types, and NOS score. Sensitivity analysis was also carried out using “leave-one-out” analysis. Egger’s test and Begg’s test were conducted to assess publication bias (33, 34). STATA software (version 12.0) was used. If existing significant publication, “trim and fill” method was applied. A *P*-value <0.05 indicated a significant difference.

## Results

**Figure 1:**
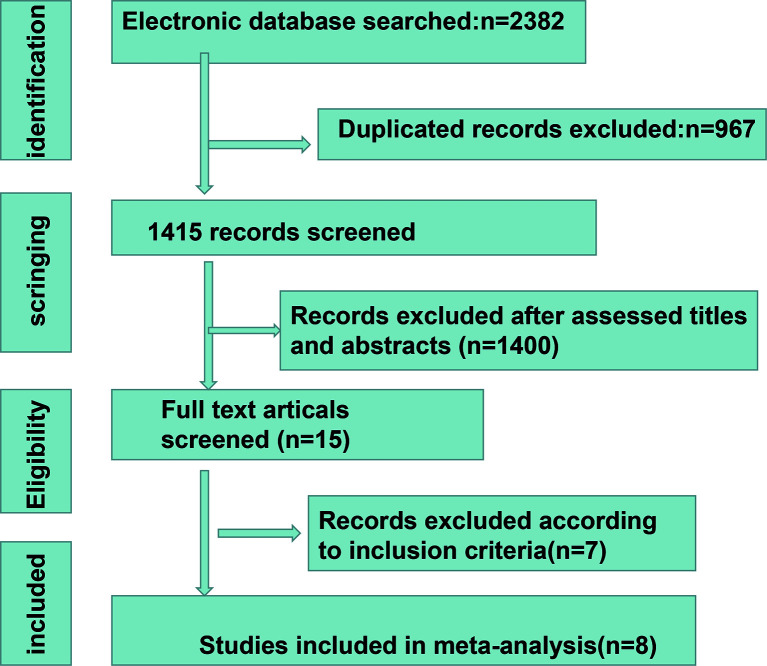
The process of study selection.

A total of 2,382 studies were retrieved from the database search. The study selection process is illustrated in **Figure 1**. Overall, 15 studies were included for full-text screening. Four studies were excluded due to a lack of related survival data (29, 35–37). One study was excluded for cases less than 20 (38). Two studies focusing on small-cell lung cancer (39) and lung carcinomas (40) were excluded. Finally, eight studies (20, 41–47) were included in this study. The information of the included studies is summarized in **Table 1** with inclusion of NOS scale for quality evaluation. The baseline patient and tumor characteristics are detailed in **Table 2**. OS was described in eight studies (20, 41–47), and PFS was mentioned in five studies (41, 43–46). The cutoff values ranged from 0.03 to 7.32 ng/ml. All studies had high NOS scores (≥7). Immunotherapy was adopted in six studies (20, 26, 41, 43–46). Five studies (20, 41, 43, 46, 47) were prospective studies, while three (42, 44, 45) studies were retrospective studies.

**Table 1:** Information on the included studies and quality assessment.

Study	Country	Study type	Sample sizes	Treatment	Outcomes	Follow-up time (M)	Quality assessment
Costantini et al. (41)	France	P	43	ICI	OS, PFS	16.3 (11.7–21.1)	8
He et al. (42)	China	R	88	Surgery	OS	67 (3–78)	8
Mazzaschi et al. (43)	Italy	P	109	ICI + C	OS, PFS	17.3	8
Murakami et al. (44)	Japan	R	233	ICI	OS, PFS	NA	7
Okuma et al. (20)	Japan	P	39	ICI	OS	NA	8
Tiako et al. (45)	France	R	51	ICI + C	OS, PFS	NA	8
Yang et al. (46)	China	P	21	ICI	OS, PFS	NA	7
Zhao et al. (47)	China	P	126	CRT	OS	NA	7

P, prospective; R, retrospective; ICI, immune checkpoint inhibitors; C, chemotherapy; CRT, chemoradiotherapy.

**Table 2:** The characteristic of included patients.

Study	Ages	Male/female	ECOG PS0-1 /over 2	Stage	Source of blood	Detection time	Cut-off value(ng/ml)
Costantini et al. (41)	68 (62-71.5)	29/14	25/18	I-IV	Plasma	Baseline or before ICI treatment	0.03
He et al. (42)	59 (36-83)	72/16	NA	Ia–IIIb	Plasma	1-2 days before surgery	3.4
Mazzaschi et al. (43)	72 (41-85)	73/36	95/14	IIIB-IV	Plasma	baseline	0.11
Murakami et al. (44)	63 (30-84)	152/81	211/22	Advanced or recurrent	Serum	Before treatment	0.09
Okuma et al. (20)	69 (50-88)	29/10	15/24	IV	Plasma	Baseline	3.36
Tiako et al. (45)	66 (60-69)	29/22	30/21	metastatic	Plasma	Baseline	0.16
Yang et al. (46)	NA	NA	NA	Advanced	Plasma	Baseline and 2 month after ICI	Fold change 0.95
Zhao et al. (47)	NA	95/31	105/21	IIIB	Plasma	Baseline, 2 and 4 weeks after treatment	0.097

### High sPD-L1 Level Is Associated With Poorer Survival Outcomes in NSCLC

**Figure 2:**
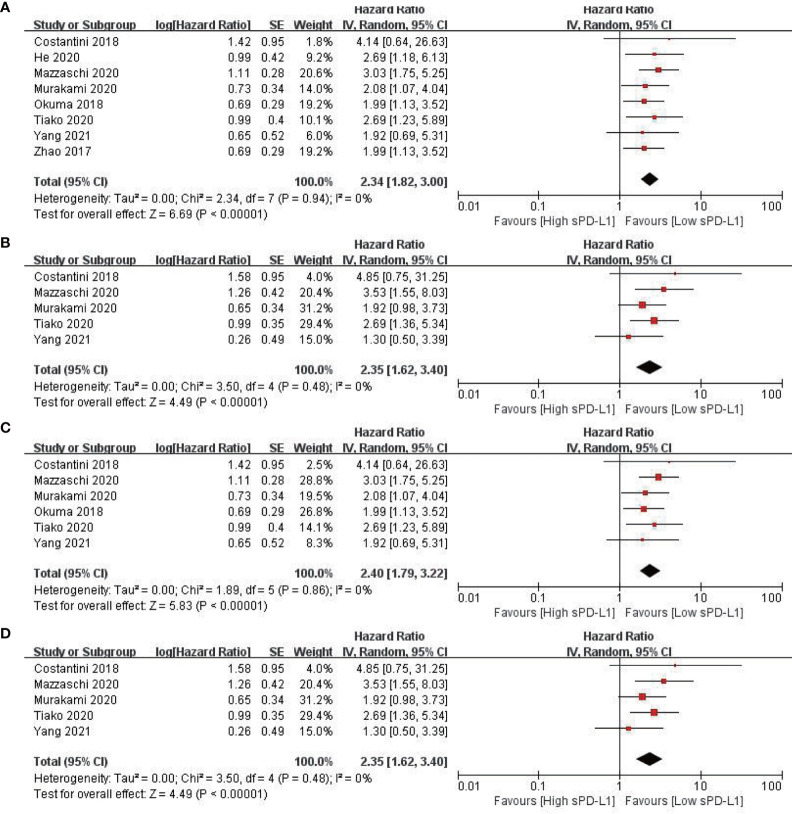
Forest plots of hazard ratio for the relationship between sPD-L1 level and survival outcomes. **(A)** Overall survival (OS) in patients with non-small cell lung cancer. **(B)** Progression-free survival (PFS) in patients with non-small cell lung cancer. **(C)** OS in patients with non-small cell lung cancer receiving immune checkpoint inhibitors. **(D)** PFS in patients with non-small cell lung cancer receiving immune checkpoint inhibitors.

All eight included studies reported OS (20, 41–47). The combined data indicated that a higher level of sPD-L1 was associated with a significantly worse OS, compared with a lower level of sPD-L1 (HR = 2.34; 95% CI, 1.82–3.00; *P* < 0.001). Moreover, there was no significant heterogeneity among the studies (*I* ^2^ = 1%; *P* = 0.43) (**Figure 2A**). By pooling the data of five studies, a higher level of sPD-L1 was found to be correlated with an unfavorable PFS (41, 43–46) using the random-effect model (HR = 2.35; 95% CI, 1.62–3.40; *P* < 0.001), with no heterogeneity (*I* ^2^ = 0, *P* = 0.48) (**Figure 2B**).

### High sPD-L1 Level Is Associated With Poorer Survival Outcomes in Patients With NSCLC Receiving Immunotherapy

Six studies (496 patients) reported the outcomes of patients receiving ICIs (20, 41, 43–46). The pooled data revealed that a higher level of sPD-L1 was related to a significantly worse OS in patients with NSCLC receiving ICI (HR = 2.34; 95% CI, 1.82–3.00; *P* < 0.001). Moreover, there was no significant heterogeneity among the studies (*I* ^2^ = 1%; *P* = 0.43) (**Figure 2C**). Furthermore, a high level of sPD-L1 was found to be correlated with an unfavorable PFS by pooling data from five studies in patients with NSCLC receiving immunotherapy (41, 43–46) using a random-effect model (HR = 2.35; 95% CI, 1.62–3.40; *P* < 0.001) (**Figure 2D**).

### Sensitivity and Subgroup Analysis

**Figure 3:**
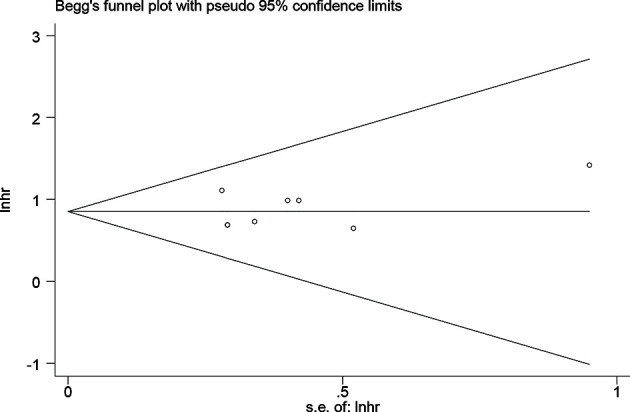
Forest plot overall survival of all patients and subgroup analysis.

A sensitivity analysis was performed to determine the stability of the findings (48). The analysis was omitted from any single study for OS at each time point. As illustrated in **Supplementary Figure S1**, the sensitivity analysis did not affect the results. Subgroup analyses were performed to confirm the reliability of the results (48). The subgroups were divided according to Asian and non-Asian populations, publication year, sample sizes, cutoff values, study types, received immunotherapy, and NOS scores. The results are presented in **Table 3** and **Figure 3**. High levels of sPD-L1 were associated with worse OS in all subgroup analyses and indicated the reliability of the results.

**Table 3:** Subgroup assessing the high sPD-L1 level and overall survival in patients with lung cancer.

Items	Number of studies	Cases	HR (95% CI)	*P*-value	Heterogeneity	
					*I* ^2^ (%)	*P*
All	8	710	2.34 (1.82, 3.00)	<0.001	0	0.94
Country						
Asia	5	507	2.09 (1.54, 2.83)	<0.001	0	0.98
Non-Asia	3	203	2.97 (1.92, 4.60)	<0.001	0	0.91
Publication year						
From 2019 onward	5	269	2.55 (1.85, 3.52)	<0.001	0	0.90
Up to 2019	3	441	2.06 (1.39, 3.05)	0.0003	0	0.75
Sample sizes						
≥100	3	468	2.37 (1.68, 3.32)	<0.001	0	0.50
<100	5	242	2.31 (1.60, 3.33)	<0.001	0	0.90
Quality scores						
≥8	5	330	2.58 (1.88, 3.55)	<0.001	0	0.85
<8	3	380	2.01 (1.35, 2.99)	<0.001	0	0.99
Study type						
Prospective	5	338	2.31 (1.70, 3.13)	<0.001	0	0.74
Retrospective	3	372	2.41 (1.57, 3,72)	<0.001	0	0.84
Source of blood						
Plasma	7	477	2.39 (1.82, 3.12)	<0.001	0	0.90
Serum	1	233	2.08 (1.07, 4.04)	0.03	–	–
Cutoff value						
≥1 ng/ml	2	127	1.64 (1.31, 2.05)	<0.001	3	0.38
<1 ng/ml	5	562	2.45 (1.80, 3.33)	<0.001	0	0.79

Moreover, in patients with NSCLC receiving ICIs, the characteristic of patients from the included studies were provided in **Table S1**. the baseline of sPD-L1 concentrations was detected in six studies (20, 41, 43–46), and five studies were reported with the OS in regard to the baseline level of sPD-L1 (20, 41, 43–45). The combined data suggested that the baseline level of sPD-L1 is a prognostic factor in patients with NSCLC receiving ICIs (**Supplementary Figure S2**).

Furthermore, age is quite associated with immunotherapy response (49) because the elderly patients will always be coupled with poor function of T cells and aggressive T cell exhaustion. In addition, we performed an analysis to determine whether age is a prognostic factor in patients with NSCLC receiving ICI. Five studies were focused on the prognostic value of age. The combined data revealed that age was not a prognostic factor for OS in patients with NSCLC receiving ICI (HR: 0.98, 95% CI: 0.94 to 1.02, *P* = 0.40) (**Supplementary Figure S3**).

**Figure 4:**
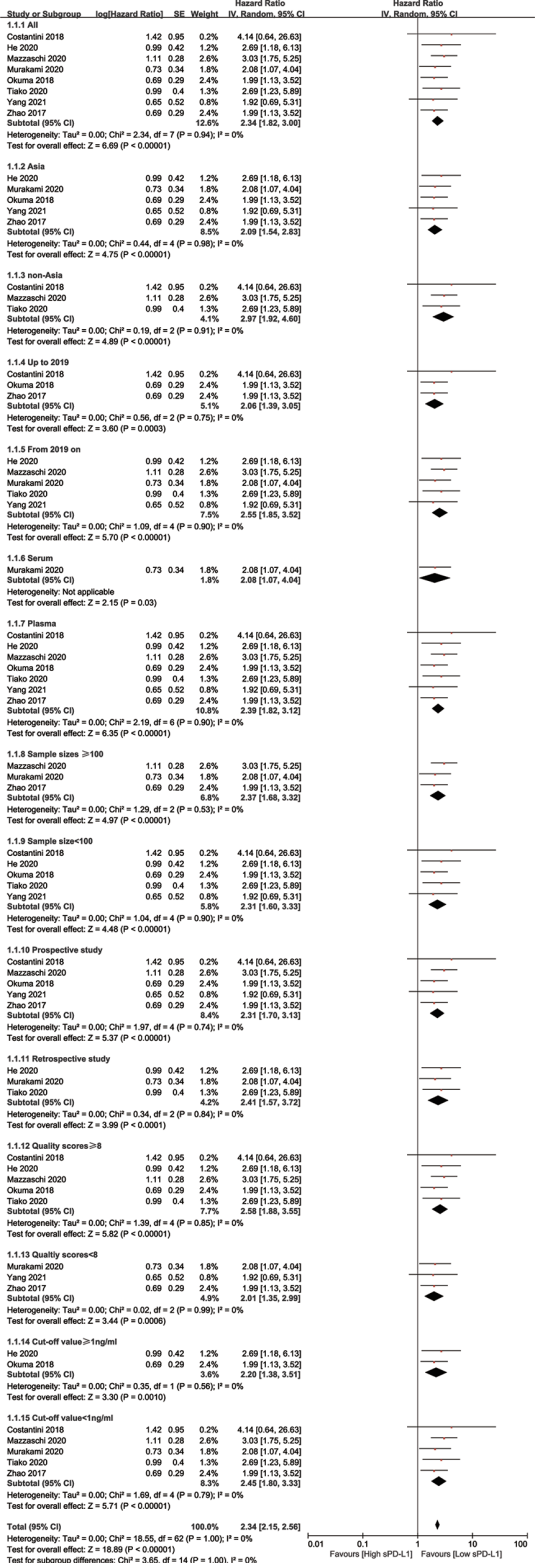
Publication bias evaluated by Begg’s test.

Publication bias was evaluated using Begg’s test and Egger’s test for OS. No potential publication bias was detected (*P* = 0.621 for Begg’s test; *P* = 0.499 for Egger’s test) (**Figure 4**). Using the funnel plot of PFS, all the studies were found to be within the 95% CI, which further confirmed that there was no potential publication bias (**Supplementary Figure S4**).

## Discussion

Based on 8 studies of 710 patients, this study demonstrated that higher levels of sPD-L1 were associated with unfavorable OS (HR = 2.34; *P* < 0.001) and PFS (HR = 2.35; *P* < 0.001) in patients with NSCLC. Moreover, the level of sPD-L1 may be considered as a prognostic marker for patients with NSCLC who received immunotherapy—poorer (HR = 2.40; *P* < 0.001). This was consistent with previous studies by Khan et al. (17) that higher sPD-L1 levels were correlated with a worse prognosis. Several studies have reported similar results, with a correlation between the high expression of sPD-L1 and poorer survival in breast cancer (50), renal cell carcinoma (21), and other solid cancers (17). Zheng et al. (29) also reported that the median OS in patients with high sPD-L1 concentrations and low sPDL-L1 levels were 18.7 and 26.8 months, respectively (*P* < 0.001). Okuma et al. (20) also reported similar results, and patients with low sPD-L1 levels had a high objective response rate. It is also important to note that enzyme-linked immunosorbent assay was the most frequently used method for measuring sPD-L1 (16), and seven of the included studies (20, 41, 43–47) used plasma. Positive results were determined based on specific receiver operating characteristic curves in most of the studies included. It was not inclusive to reach a consensus on the cutoff of positive from normal controls. Cheng et al. reported that the plasma sPD-L1 levels were different according to pathological types (51). Jin et al. (39) reported that the sPD-L1 levels were lower in healthy controls than in patients with lung cancer (1.2 *vs*. 7 ng/ml). He et al. (42) also described that the mean sPD-L1 concentrations in patients with NSCLC and healthy volunteers were 3.84 and 0.79 ng/mL, respectively. Thus, there is an urgent need to determine the optimal cutoff value in the future based on large case studies.

Six studies (496 patients) (20, 41, 43–46) reported results focusing on patients with NSCLC treated with immunotherapy. Costantini et al. (41) indicated that patients with low sPD-L1 concentrations were likely to benefit from immunotherapy. A study reported by Okuma et al. (20), including 39 patients with NSCLC treated with nivolumab, indicated that OS was significantly reduced in patients with high sPD-L1 levels than in those with low sPD-L1 levels. A recent study of 51 nivolumab-treated patients with NSCLC revealed that the baseline sPD-L1 levels were related to poor survival outcomes (45). Another study (43) with 109 patients with NSCLC received immunotherapy suggested that the median OS was 5.8 and 15.0 months in high and low levels of sPD-L1 patients, respectively. Murakami et al. (44) reported a study of 233 patients with NSCLC treated with immunotherapy revealed that the PFS and OS in the low-sPD-L1 group were longer than those in the high-sPD-L1 group. In the study of Okuma et al. (20), it was noted that high sPD-L1 levels in patients were correlated with a shorter time to treatment failure compared with those patients with low sPD-L1 levels. The objective response rate was favorable to the low-plasma-sPD-L1-concentration group (20). Similar results were found in patients with gastric cancer receiving ICIs (52). This pooled data of the six included studies indicated that sPD-L1 had a prognostic role in patients with NSCLC treated with immunotherapy (HR = 2.40; *P* < 0.001). This is consistent with a previous study showing that the sPD-L1 levels can be a prognostic marker in patients with melanoma receiving ICIs (53). Moreover, monitoring the level of sPD-L1 may be helpful for predicting survival in patients with cancer and subsequently improving the treatment effect (14).

An open question that remains to be answered is what makes sPD-L1 a suitable prognostic marker for cancer. The biology rationale is not clear to us. A potential hypothesis is that the inhibition of sPD-L1 can result in a similar function to other checkpoint inhibitors, thereby achieving a checkpoint inhibitor effect. Some studies reported that the inhibition of sPD-L1 restricting tumor growth showed a similar mechanism to that of anti-PD-L1 in mAb-injected mice (54, 55). A future study is needed. Secondly, the sPD-L1 levels were mostly tested at baseline. No dynamic analysis was carried out in the majority of patients. The sPD-L1 level at baseline was also a prognostic factor in patients with NSCLC receiving ICIs. The variation of sPD-L1 was reported between baseline and after 2 and 4 weeks of radiotherapy in the study of Zhao et al., which indicated a reduction of sPD-L1 after radiotherapy and patients with low baseline sPD-L1 concentrations reached a longer OS than those with higher sPD-L1 concentrations (47). Costantini et al. (41) indicated that there was no statistical difference in sPD-L1 levels between responders and non-responders to ICIs. High sPDL-L1 levels at baseline and an increase in sPD-L1 levels were correlated with poor survival outcomes (OS and PFS). In the study by He et al. (42), the detection time of sPD-L1 was 1 to 2 days before surgery.

The relationship between the PD-L1 expression of the tissue and the level of sPD-L1 is not fully understood. As described in the study of Mazzachi et al. (43), there was no significant correlation between sPD-L1 level and the expression of tissue PD-L1 assessed on primary tumors. Murakami et al. (44) reported the expression of tissue PD-L1 and sPD-L1 level, but the details of the two parameters were not shown. In the study of Costantini et al. (41), no correlation was found between sPD-L1 concentrations and the expression of PD-L1 in immunohistochemistry performed on the initial biopsy. In contrast, Yang et al. revealed that the blood PD-L1 had a significant positive correlation with tissue PD-L1 expression at the same time points. More studies are needed to explore these topics.

As described by He et al. (42), there was no association between sPD-L1 levels and clinicopathologic features (sex, histologic type, differentiation degree, T stage, N stage, tumor size, pTNM stage, and smoking status) in patients with NSCLC. Moreover, Murakami et al. (44) also indicated that there was no significant correlation between sPD-L1 concentrations and clinicopathologic characteristics, including age, sex, Eastern Cooperative Oncology Group performance status, smoking status, histology, brain metastasis, and *EGFR* mutations. However, sPD-L1 concentrations were associated with live metastasis (*P* = 0.015). Mazzachi et al. (43) also indicated that sPD-L1 concentrations were not associated with sex, age, smoking status, or histology. However, the authors indicated that sPD-L1 levels were related to N metastatic sites and live metastasis, and another study indicated that the sPD-L1 levels were related to abdominal organ metastasis (29). Some researchers have revealed that there may be a relationship between high lung cancer tumor burden and the elevated sPD-L1 levels in patients with NSCLC (56). Okuma et al. (40) also investigated the relationship between sPD-L1 levels and the clinical features and revealed that the sPD-L1 levels were not related to the clinicopathological features in patients with advanced lung cancer. Due to the limited data and studies, combined data studies were not performed.

The relationship between age and immunotherapy response in patients receiving ICIs is controversial. In a clinical study, subgroup analyses (≥65 *vs*. <65 years old) suggested no significant difference in survival outcomes (57). A study reported that patients with advancing age with NSCLC and receiving ICIs seem to have a longer PFS (58). In contrast, a study published in 2015 indicated that elderly patients have a shorter OS (59). A meta-analysis comparing the efficacy of immunotherapy in elderly *vs*. young populations indicated that OS was not significant between the two groups (HR = 0.76, *P* = 0.66) (60). This is in consistent with our analysis. More studies are required to evaluate the effect of age on immunotherapy.

This study had some limitations. First, some of the included studies were retrospective studies, and there may have been a selection bias or publication bias, as positive results are more easily published in journals compared with negative results. Second, in regard to heterogeneity, all analyses used the random-effect model (61). In the process of evaluation of the results from different studies, the heterogeneity among these studies should be taken into consideration. Heterogeneity could have come from study design, different stages, different management, different detection method, sample sizes, or ages. Moreover, sensitivity and subgroups analyses were performed to identify the potential source of heterogeneity. Our analyses indicated that all the analyses were with low heterogeneity and indicated the reliability of the results. Moreover, the cutoff values, the definitions of abnormal high level, and the evaluation methods for sPD-L1 and high PD-L1 were not consistent, all of which may have contributed to heterogeneity, and the cutoff values of sPD-L1 were not uniform, leading to limitations in clinical applications (25). However, all of the analyses in this meta-analysis had low heterogeneity, and the analyses used a random-effect model, where the results were reliable. Finally, due to the limited data and small number of patients in a few studies, we were unable to determine the relationship between sPD-L1 concentrations and the clinical features due to an inability to pool the data together.

In conclusion, our meta-analysis indicates that sPD-L1 has a prognostic role in patients with NSCLC. Moreover, low sPD-L1 levels may be a prognostic factor in patients receiving immunotherapy. A high expression of sPD-L1 was correlated significantly with worse OS and PFS. Prospective studies with well-designed and standard assessment methods (41) should be carried out in the future to determine the prognostic role of sPD-L1 in NSCLC.

## Data Availability Statement

The original contributions presented in the study are included in the article/**Supplementary Material**. Further inquiries can be directed to the corresponding authors.

## Author Contributions

GL and ZZ contributed to the study design and manuscript drafting. GL and F-MK contributed to editing and proving. All authors contributed to the article and approved the submitted version.

## Funding

This study was supported by the Natural Science Foundation of Shenzhen (no. JCYJ20170307095828424). China NSF8187110989 as well as Shenzhen Science and Technology Commission (Grant No: KQTD20180411185028798).

## Conflict of Interest

The authors declare that the research was conducted in the absence of any commercial or financial relationships that could be construed as a potential conflict of interest.

## Publisher’s Note

All claims expressed in this article are solely those of the authors and do not necessarily represent those of their affiliated organizations, or those of the publisher, the editors and the reviewers. Any product that may be evaluated in this article, or claim that may be made by its manufacturer, is not guaranteed or endorsed by the publisher.

## Supplementary Material

Supplementary Figure 1 Sensitivity analysis by omitting every single study.

Supplementary Figure 2 Forest plot overall survival in regard to the baseline level of sPD-L1 in patients with non-small cell lung cancer receiving immune checkpoint inhibitors.

Supplementary Figure 3 Forest plot overall survival in regard to age in patients with non-small cell lung cancer receiving immune checkpoint inhibitors.

Supplementary Figure 4 Funnel plot of progression-free survival in patients with non-small cell lung cancer.

## References

[1] Bray F Ferlay J Soerjomataram I Siegel RL Torre LA Jemal A . Global Cancer Statistics 2018: GLOBOCAN Estimates of Incidence and Mortality Worldwide for 36 Cancers in 185 Countries. CA Cancer J Clin (2018) 68:394–424. doi: 10.3322/caac.21492 30207593

[2] El OB Behera M Kim S Berry LD Sica G Pillai RN . Characteristics and Outcomes of Patients With Metastatic KRAS-Mutant Lung Adenocarcinomas: The Lung Cancer Mutation Consortium Experience. J Thorac Oncol (2019) 14:876–89. doi: 10.1016/j.jtho.2019.01.020 PMC8108452 30735816

[3] Gordon SR Maute RL Dulken BW Hutter G George BM McCracken MN . PD-1 Expression by Tumour-Associated Macrophages Inhibits Phagocytosis and Tumour Immunity. Nature (2017) 545:495–9. doi: 10.1038/nature22396 PMC5931375 28514441

[4] Reck M Rodriguez-Abreu D Robinson AG Hui R Csoszi T Fulop A . Pembrolizumab Versus Chemotherapy for PD-L1-Positive Non-Small-Cell Lung Cancer. N Engl J Med (2016) 375:1823–33. doi: 10.1056/NEJMoa1606774 27718847

[5] Sun C Mezzadra R Schumacher TN . Regulation and Function of the PD-L1 Checkpoint. Immunity (2018) 48:434–52. doi: 10.1016/j.immuni.2018.03.014 PMC7116507 29562194

[6] Cui S Su X Dong L Qian J Ye L Zhang T . Programmed Cell Death Ligand 1 Protein Levels Predicted Survival of Non-Small Cell Lung Cancer. J Cancer (2017) 8:4075–82. doi: 10.7150/jca.21415 PMC5706010 29187883

[7] Ishii H Azuma K Kawahara A Yamada K Imamura Y Tokito T . Significance of Programmed Cell Death-Ligand 1 Expression and its Association With Survival in Patients With Small Cell Lung Cancer. J Thorac Oncol (2015) 10:426–30. doi: 10.1097/JTO.0000000000000414 25384063

[8] Gray JE Okamoto I Sriuranpong V Vansteenkiste J Imamura F Lee JS . Tissue and Plasma EGFR Mutation Analysis in the FLAURA Trial: Osimertinib Versus Comparator EGFR Tyrosine Kinase Inhibitor as First-Line Treatment in Patients With EGFR-Mutated Advanced Non-Small Cell Lung Cancer. Clin Cancer Res (2019) 25:6644–52. doi: 10.1158/1078-0432.CCR-19-1126 PMC7209579 31439584

[9] Wislez M Domblides C Greillier L Mazieres J Monnet I Kiakouama-Maleka L . Circulating Tumor DNA in Advanced Non-Small-Cell Lung Cancer Patients With HIV is Associated With Shorter Overall Survival: Results From a Phase II Trial (IFCT-1001 CHIVA). Lung Cancer (2021) 157:124–30. doi: 10.1016/j.lungcan.2021.05.013 34016488

[10] Duchemann B Remon J Naigeon M Mezquita L Ferrara R Cassard L . Integrating Circulating Biomarkers in the Immune Checkpoint Inhibitor Treatment in Lung Cancer. Cancers (Basel) (2020) 12:3625. doi: 10.3390/cancers12123625 PMC7761725 33287347

[11] Leon-Flores A Del REP Alvarez-Garcia LX Piten-Isidro E Reyes-Teran G . Increased Levels of Soluble Co-Stimulatory Molecule PD-L1 (B7-H1) in the Plasma of Viraemic HIV-1(+) Individuals. Immunol Lett (2018) 203:70–9. doi: 10.1016/j.imlet.2018.09.007 30236481

[12] Daassi D Mahoney KM Freeman GJ . The Importance of Exosomal PDL1 in Tumour Immune Evasion. Nat Rev Immunol (2020) 20:209–15. doi: 10.1038/s41577-019-0264-y 31965064

[13] Chang B Huang T Wei H Shen L Zhu D He W . The Correlation and Prognostic Value of Serum Levels of Soluble Programmed Death Protein 1 (sPD-1) and Soluble Programmed Death-Ligand 1 (sPD-L1) in Patients With Hepatocellular Carcinoma. Cancer Immunol Immunother (2019) 68:353–63. doi: 10.1007/s00262-018-2271-4 PMC6426820 30506460

[14] Fu R Jing CQ Li XR Tan ZF Li HJ . Prognostic Significance of Serum PD-L1 Level in Patients With Locally Advanced or Metastatic Esophageal Squamous Cell Carcinoma Treated With Combination Cytotoxic Chemotherapy. Cancer Manag Res (2021) 13:4935–46. doi: 10.2147/CMAR.S312690 PMC8232859 34188546

[15] Ha H Nam AR Bang JH Park JE Kim TY Lee KH . Soluble Programmed Death-Ligand 1 (Spdl1) and Neutrophil-to-Lymphocyte Ratio (NLR) Predicts Survival in Advanced Biliary Tract Cancer Patients Treated With Palliative Chemotherapy. Oncotarget (2016) 7:76604–12. doi: 10.18632/oncotarget.12810 PMC5363533 27780932

[16] Abu HT Furqan M Ballas Z Clamon G . The Clinical Significance of Soluble PD-1 and PD-L1 in Lung Cancer. Crit Rev Oncol Hematol (2019) 143:148–52. doi: 10.1016/j.critrevonc.2019.08.009 31675543

[17] Khan M Zhao Z Arooj S Fu Y Liao G . Soluble PD-1: Predictive, Prognostic, and Therapeutic Value for Cancer Immunotherapy. Front Immunol (2020) 11:587460. doi: 10.3389/fimmu.2020.587460 33329567 PMC7710690

[18] Frigola X Inman BA Lohse CM Krco CJ Cheville JC Thompson RH . Identification of a Soluble Form of B7-H1 That Retains Immunosuppressive Activity and Is Associated With Aggressive Renal Cell Carcinoma. Clin Cancer Res (2011) 17:1915–23. doi: 10.1158/1078-0432.CCR-10-0250 PMC3241002 21355078

[19] Liu C Jiang J Gao L Wang X Hu X Wu M . Soluble PD-1 Aggravates Progression of Collagen-Induced Arthritis Through Th1 and Th17 Pathways. Arthritis Res Ther (2015) 17:340. doi: 10.1186/s13075-015-0859-z 26608464 PMC4659197

[20] Okuma Y Wakui H Utsumi H Sagawa Y Hosomi Y Kuwano K . Soluble Programmed Cell Death Ligand 1 as a Novel Biomarker for Nivolumab Therapy for Non-Small-Cell Lung Cancer. Clin Lung Cancer (2018) 19:410–7. doi: 10.1016/j.cllc.2018.04.014 29859759

[21] Larrinaga G Solano-Iturri JD Errarte P Unda M Loizaga-Iriarte A Perez-Fernandez A . Soluble PD-L1 Is an Independent Prognostic Factor in Clear Cell Renal Cell Carcinoma. Cancers (Basel) (2021) 13:667. doi: 10.3390/cancers13040667 33562338 PMC7915750

[22] Nukui A Masuda A Abe H Arai K Yoshida KI Kamai T . Increased Serum Level of Soluble Interleukin-2 Receptor is Associated With a Worse Response of Metastatic Clear Cell Renal Cell Carcinoma to Interferon Alpha and Sequential VEGF-Targeting Therapy. BMC Cancer (2017) 17:372. doi: 10.1186/s12885-017-3369-3 28545581 PMC5445282

[23] Wang Q Zhang J Tu H Liang D Chang DW Ye Y . Soluble Immune Checkpoint-Related Proteins as Predictors of Tumor Recurrence, Survival, and T Cell Phenotypes in Clear Cell Renal Cell Carcinoma Patients. J Immunother Cancer (2019) 7:334. doi: 10.1186/s40425-019-0810-y 31783776 PMC6884764

[24] Asanuma K Nakamura T Hayashi A Okamoto T Iino T Asanuma Y . Soluble Programmed Death-Ligand 1 Rather Than PD-L1 on Tumor Cells Effectively Predicts Metastasis and Prognosis in Soft Tissue Sarcomas. Sci Rep (2020) 10:9077. doi: 10.1038/s41598-020-65895-0 32493964 PMC7270095

[25] Huang P Hu W Zhu Y Wu Y Lin H . The Prognostic Value of Circulating Soluble Programmed Death Ligand-1 in Cancers: A Meta-Analysis. Front Oncol (2020) 10:626932. doi: 10.3389/fonc.2020.626932 33718120 PMC7950317

[26] Li X Zheng Y Yue F . Prognostic Value of Soluble Programmed Cell Death Ligand-1 (sPD-L1) in Various Cancers: A Meta-Analysis. Target Oncol (2021) 16:13–26. doi: 10.1007/s11523-020-00763-5 33222017

[27] Wei W Xu B Wang Y Wu C Jiang J Wu C . Prognostic Significance of Circulating Soluble Programmed Death Ligand-1 in Patients With Solid Tumors: A Meta-Analysis. Med (Baltimore) (2018) 97:e9617. doi: 10.1097/MD.0000000000009617 PMC5779759 29504990

[28] Zheng Z Bu Z Liu X Zhang L Li Z Wu A . Level of Circulating PD-L1 Expression in Patients With Advanced Gastric Cancer and its Clinical Implications. Chin J Cancer Res (2014) 26:104–11. doi: 10.3978/j.issn.1000-9604.2014.02.08. PMC3937742 24653632

[29] Zhang J Gao J Li Y Nie J Dai L Hu W . Circulating PD-L1 in NSCLC Patients and the Correlation Between the Level of PD-L1 Expression and the Clinical Characteristics. Thorac Cancer (2015) 6:534–8. doi: 10.1111/1759-7714.12247 PMC4511334 26273411

[30] Moher D Liberati A Tetzlaff J Altman DG . Preferred Reporting Items for Systematic Reviews and Meta-Analyses: The PRISMA Statement. BMJ (2009) 339:b2535. doi: 10.1136/bmj.b2535. 19622551 PMC2714657

[31] Zhao Z Liao G Li Y Zhou S Zou H Fernando S . Prognostic Value of Carbonic Anhydrase IX Immunohistochemical Expression in Renal Cell Carcinoma: A Meta-Analysis of the Literature. PloS One (2014) 9:e114096. doi: 10.1371/journal.pone.0114096 25426861 PMC4245260

[32] Higgins JP Altman DG Gotzsche PC Juni P Moher D Oxman AD . The Cochrane Collaboration's Tool for Assessing Risk of Bias in Randomised Trials. BMJ (2011) 343:d5928. doi: 10.1136/bmj.d5928 22008217 PMC3196245

[33] Begg CB Mazumdar M . Operating Characteristics of a Rank Correlation Test for Publication Bias. Biometrics (1994) 50:1088–101. doi: 10.2307/2533446 7786990

[34] Egger M Davey SG Schneider M Minder C . Bias in Meta-Analysis Detected by a Simple, Graphical Test. BMJ (1997) 315:629–34. doi: 10.1136/bmj.315.7109.629 PMC2127453 9310563

[35] Castello A Rossi S Toschi L Mansi L Lopci E . Soluble PD-L1 in NSCLC Patients Treated With Checkpoint Inhibitors and Its Correlation With Metabolic Parameters. Cancers (Basel) (2020) 12:1373. doi: 10.3390/cancers12061373 PMC7352887 32471030

[36] Matsuo N Azuma K Hattori S Ohtake J Kawahara A Ishii H . Association Between Soluble Immune Mediators and Tumor Responses in Patients With Nonsmall Cell Lung Cancer Treated With Anti-PD-1 Inhibitor. Int J Cancer (2019) 144:1170–9. doi: 10.1002/ijc.31923 30307035

[37] Jovanovic D Roksandic-Milenkovic M Kotur-Stevuljevic J Ceriman V Vukanic I Samardzic N . Soluble sPD-L1 and Serum Amyloid A1 as Potential Biomarkers for Lung Cancer. J Med Biochem (2019) 38:332–41. doi: 10.2478/jomb-2018-0036 PMC6534957 31156344

[38] Ando K Hamada K Watanabe M Ohkuma R Shida M Onoue R . Plasma Levels of Soluble PD-L1 Correlate With Tumor Regression in Patients With Lung and Gastric Cancer Treated With Immune Checkpoint Inhibitors. Anticancer Res (2019) 39:5195–201. doi: 10.21873/anticanres.13716 31519633

[39] Jin J Si J Liu Y Wang H Ni R Wang J . Elevated Serum Soluble Programmed Cell Death Ligand 1 Concentration as a Potential Marker for Poor Prognosis in Small Cell Lung Cancer Patients With Chemotherapy. Respir Res (2018) 19:197. doi: 10.1186/s12931-018-0885-x 30290817 PMC6173911

[40] Okuma Y Hosomi Y Nakahara Y Watanabe K Sagawa Y Homma S . High Plasma Levels of Soluble Programmed Cell Death Ligand 1 are Prognostic for Reduced Survival in Advanced Lung Cancer. Lung Cancer (2017) 104:1–6. doi: 10.1016/j.lungcan.2016.11.023 28212990

[41] Costantini A Julie C Dumenil C Helias-Rodzewicz Z Tisserand J Dumoulin J . Predictive Role of Plasmatic Biomarkers in Advanced Non-Small Cell Lung Cancer Treated by Nivolumab. Oncoimmunology (2018) 7:e1452581. doi: 10.1183/13993003.congress-2018.OA3302 30221046 PMC6136870

[42] He J Pan Y Guo Y Li B Tang Y . Study on the Expression Levels and Clinical Significance of PD-1 and PD-L1 in Plasma of NSCLC Patients. J Immunother (2020) 43:156–64. doi: 10.1097/CJI.0000000000000315 32168233

[43] Mazzaschi G Minari R Zecca A Cavazzoni A Ferri V Mori C . Soluble PD-L1 and Circulating CD8+PD-1+ and NK Cells Enclose a Prognostic and Predictive Immune Effector Score in Immunotherapy Treated NSCLC Patients. Lung Cancer (2020) 148:1–11. doi: 10.1016/j.lungcan.2020.07.028 32768804

[44] Murakami S Shibaki R Matsumoto Y Yoshida T Goto Y Kanda S . Association Between Serum Level Soluble Programmed Cell Death Ligand 1 and Prognosis in Patients With Non-Small Cell Lung Cancer Treated With Anti-PD-1 Antibody. Thorac Cancer (2020) 11:3585–95. doi: 10.1111/1759-7714.13721 PMC7705908 33108686

[45] Tiako MM Jouinot A Giroux-Leprieur E Fabre E Wislez M Alifano M . Predictive Value of Soluble PD-1, PD-L1, VEGFA, CD40 Ligand and CD44 for Nivolumab Therapy in Advanced Non-Small Cell Lung Cancer: A Case-Control Study. Cancers (Basel) (2020) 12:473. doi: 10.3390/cancers12020473 PMC7072584 32085544

[46] Yang Q Chen M Gu J Niu K Zhao X Zheng L . Novel Biomarkers of Dynamic Blood PD-L1 Expression for Immune Checkpoint Inhibitors in Advanced Non-Small-Cell Lung Cancer Patients. Front Immunol (2021) 12:665133. doi: 10.3389/fimmu.2021.665133 33936103 PMC8085403

[47] Zhao J Zhang P Wang J Xi Q Zhao X Ji M . Plasma Levels of Soluble Programmed Death Ligand-1 may be Associated With Overall Survival in Nonsmall Cell Lung Cancer Patients Receiving Thoracic Radiotherapy. Med (Baltimore) (2017) 96:e6102. doi: 10.1097/MD.0000000000006102 PMC5319514 28207525

[48] Liao G Zhao Z Yang H Chen M Li X . Can Prognostic Nutritional Index be a Prediction Factor in Esophageal Cancer?: A Meta-Analysis. Nutr Cancer (2020) 72:187–93. doi: 10.1080/01635581.2019.1631859 31272238

[49] Grosjean H Dolter S Meyers DE Ding PQ Stukalin I Goutam S . Effectiveness and Safety of First-Line Pembrolizumab in Older Adults With PD-L1 Positive Non-Small Cell Lung Cancer: A Retrospective Cohort Study of the Alberta Immunotherapy Database. Curr Oncol (2021) 28:4213–22. doi: 10.3390/curroncol28050357 PMC8534423 34677275

[50] Han B Dong L Zhou J Yang Y Guo J Xuan Q . The Clinical Implication of Soluble PD-L1 (sPD-L1) in Patients With Breast Cancer and its Biological Function in Regulating the Function of T Lymphocyte. Cancer Immunol Immunother (2021) 70:2893–909. doi: 10.1007/s00262-021-02898-4 PMC8423647 33688997

[51] Cheng S Zheng J Zhu J Xie C Zhang X Han X . PD-L1 Gene Polymorphism and High Level of Plasma Soluble PD-L1 Protein may be Associated With Non-Small Cell Lung Cancer. Int J Biol Markers (2015) 30:e364–8. doi: 10.5301/jbm.5000170 26349666

[52] Shigemori T Toiyama Y Okugawa Y Yamamoto A Yin C Narumi A . Soluble PD-L1 Expression in Circulation as a Predictive Marker for Recurrence and Prognosis in Gastric Cancer: Direct Comparison of the Clinical Burden Between Tissue and Serum PD-L1 Expression. Ann Surg Oncol (2019) 26:876–83. doi: 10.1245/s10434-018-07112-x 30565045

[53] Zhou J Mahoney KM Giobbie-Hurder A Zhao F Lee S Liao X . Soluble PD-L1 as a Biomarker in Malignant Melanoma Treated With Checkpoint Blockade. Cancer Immunol Res (2017) 5:480–92. doi: 10.1158/2326-6066.CIR-16-0329 PMC5642913 28522460

[54] He YF Zhang GM Wang XH Zhang H Yuan Y Li D . Blocking Programmed Death-1 Ligand-PD-1 Interactions by Local Gene Therapy Results in Enhancement of Antitumor Effect of Secondary Lymphoid Tissue Chemokine. J Immunol (2004) 173:4919–28. doi: 10.4049/jimmunol.173.8.4919 15470033

[55] Yuan Y He Y Wang X Zhang H Li D Feng Z . Investigation on the Effects of Soluble Programmed Death-1 (sPD-1) Enhancing Anti-Tumor Immune Response. J Huazhong Univ Sci Technolog Med Sci (2004) 24:531–4. doi: 10.1007/BF02911345 15791831

[56] Vecchiarelli S Passiglia F D'Incecco A Gallo M De Luca A Rossi E . Circulating Programmed Death Ligand-1 (cPD-L1) in Non-Small-Cell Lung Cancer (NSCLC). Oncotarget (2018) 9:17554–63. doi: 10.18632/oncotarget.24785 PMC5915137 29707129

[57] Paz-Ares L Luft A Vicente D Tafreshi A Gumus M Mazieres J . Pembrolizumab Plus Chemotherapy for Squamous Non-Small-Cell Lung Cancer. N Engl J Med (2018) 379:2040–51. doi: 10.1056/NEJMoa1810865 30280635

[58] Lichtenstein M Nipp RD Muzikansky A Goodwin K Anderson D Newcomb RA . Impact of Age on Outcomes With Immunotherapy in Patients With Non-Small Cell Lung Cancer. J Thorac Oncol (2019) 14:547–52. doi: 10.1016/j.jtho.2018.11.011 30476576

[59] Borghaei H Paz-Ares L Horn L Spigel DR Steins M Ready NE . Nivolumab Versus Docetaxel in Advanced Nonsquamous Non-Small-Cell Lung Cancer. N Engl J Med (2015) 373:1627–39. doi: 10.1056/NEJMoa1507643 PMC5705936 26412456

[60] Ciccarese C Iacovelli R Bria E Palazzo A Maiorano BA Mosillo C . The Anticancer Efficacy of Immune Checkpoint Inhibitors According to Patients' Age: A Systematic Review and Meta-Analysis. J Immunother (2020) 43:95–103. doi: 10.1097/CJI.0000000000000312 32080018

[61] Knapp G Biggerstaff BJ Hartung J . Assessing the Amount of Heterogeneity in Random-Effects Meta-Analysis. Biom J (2006) 48:271–85. doi: 10.1002/bimj.200510175 16708778

