# Supplementary figures and images for: Prognostic Role of Soluble Programmed Death Ligand 1 in Non-Small Cell Lung Cancer: A Systematic Review and Meta-Analysis
Source: Front Oncol. 2021 Dec 23;11:774131. doi: 10.3389/fonc.2021.774131 (PMC8732757; doi:10.3389/fonc.2021.774131)

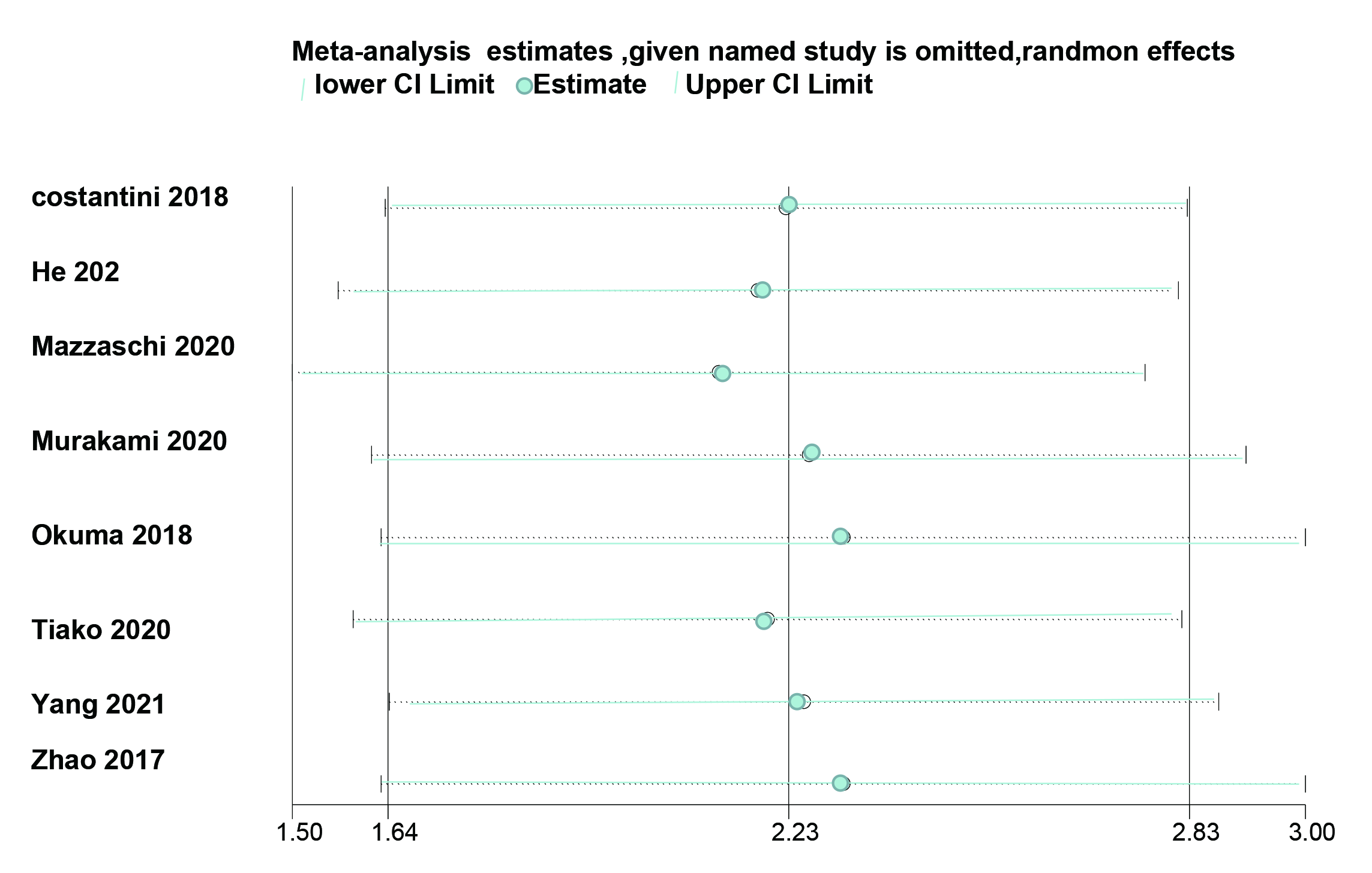

Supplement: Supplementary Figure 1 — Sensitivity analysis by omitting every single study. [file Image_1.tif]

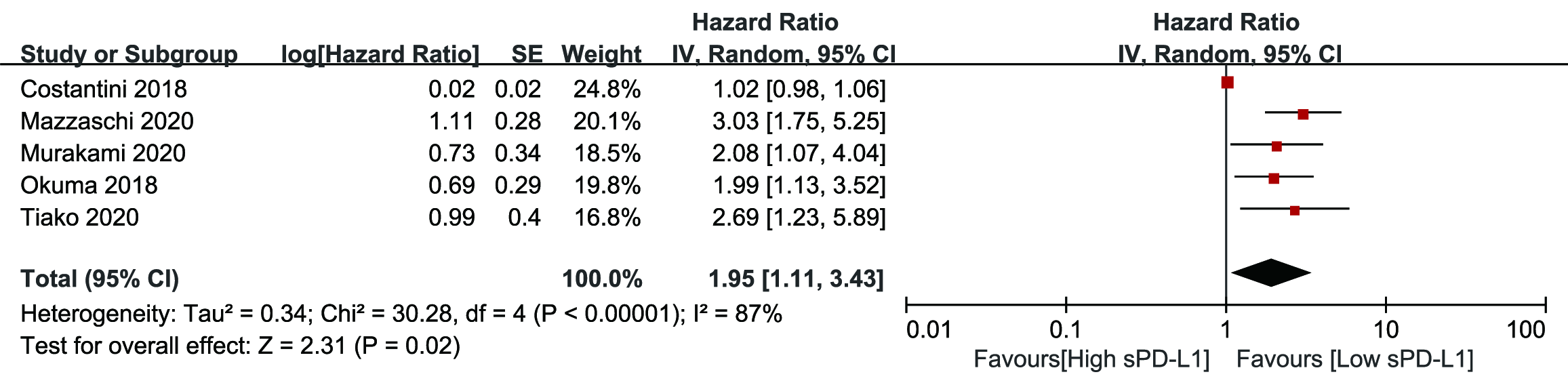

Supplement: Supplementary Figure 2 — Forest plot overall survival in regard to the baseline level of sPD-L1 in patients with non-small cell lung cancer receiving immune checkpoint inhibitors. [file Image_2.tif]

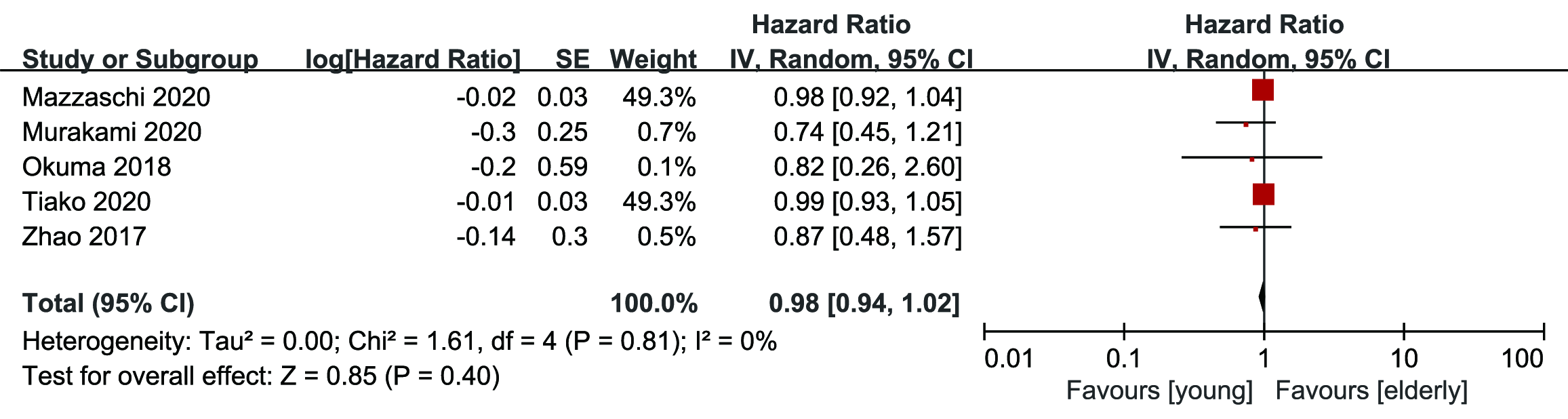

Supplement: Supplementary Figure 3 — Forest plot overall survival in regard to age in patients with non-small cell lung cancer receiving immune checkpoint inhibitors. [file Image_3.tif]

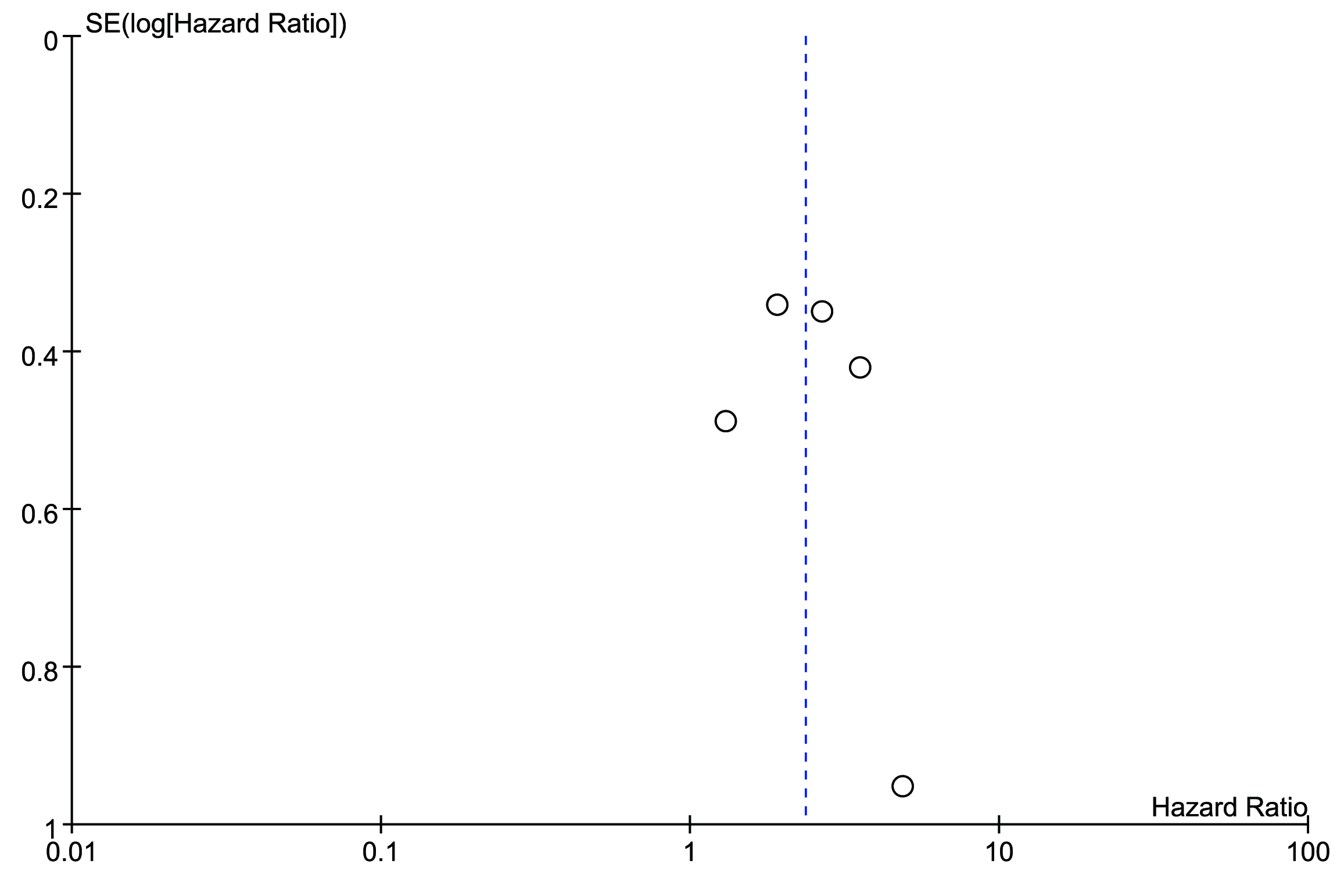

Supplement: Supplementary Figure 4 — Funnel plot of progression-free survival in patients with non-small cell lung cancer. [file Image_4.tif]
