# Supplementary material for: Prognostic Role of Soluble Programmed Death Ligand 1 in Non-Small Cell Lung Cancer: A Systematic Review and Meta-Analysis
Source: Front Oncol. 2021 Dec 23;11:774131. doi: 10.3389/fonc.2021.774131 (PMC8732757; doi:10.3389/fonc.2021.774131)
Supplement: Supplementary file 5 [file Table_1.docx]

| Study | Ages | Male/female | ECOG PS  0-1 /over 2 | Stage | Histotype(ADC/SCC/others) | Smoking history(Yes/No) | ICI treatment(I line/II line/≥ III line) | PD-L1 TPS  < 1%/1% to 49%/≥ 50% |
| --- | --- | --- | --- | --- | --- | --- | --- | --- |
| Costantini  2018 | 68(62-71.5) | 29/14 | 25/18 | I-IV | 28/9/6 | 38/5 | 0/29/14 | NA |
| Mazzaschi  2020 | 72(41-85) | 73/36 | 95/14 | IIIB-IV | 70/32/7 | 84/25 | 15/73/21 | NA |
| Murakami  2020 | 63(30-84) | 152/81 | 211/22 | Advanced or recurrent | 181/52^a^ | 201/32 | 40/193^b^ | 33/44/79 |
| Okuma 2018 | 69(50-88) | 29/10 | 15/24 | IV | 28/7/4 | 28/11^c^ | 32/7^b^ | NA |
| Tiako  2020 | 66(60-69) | 29/22 | 30/21 | Metastatic | 40/11/0 | 49/2 | 0/35/16 | 14/17/9 |
| Yang 2021 | NA | NA | NA | Advanced | NA | NA | NA | NA |

Table S1 The characteristic of patients with non-small cell lung cancer receiving immunotherapy

ECOG PS: Eastern Cooperative Oncology Group performance status;ICI:immune checkpoint inhibitors;SCC: Squamous Cell Carcinoma; ADC: Adenocarcinoma;

NA:not available; a,non-Squamous-cell carcinoma / Squamous-cell carcinoma;b, I line/ ≥ II line;c ,heavy smokers/light smokers
